# Trends in Opioid Use Disorder in the Veterans Health Administration, 2005-2022

**DOI:** 10.1001/jamanetworkopen.2024.51821

**Published:** 2024-12-20

**Authors:** Lauren R. Gorfinkel, Carol A. Malte, David S. Fink, Zachary L. Mannes, Melanie M. Wall, Mark Olfson, Ofir Livne, Salomeh Keyhani, Katherine M. Keyes, Silvia S. Martins, Magdalena Cerdá, Sarah Gutkind, Charles C. Maynard, Andrew J. Saxon, Tracy Simpson, Gregg Gonsalves, Haidong Lu, Yoanna McDowell, Deborah S. Hasin

**Affiliations:** 1 Department of Psychiatry, Columbia University Irving Medical Center, New York, New York; 2 New York State Psychiatric Institute, New York; 3 Health Services Research and Development, Seattle Center of Innovation for Veteran-Centered and Value-Driven Care, Veterans Affairs (VA) Puget Sound Health Care System, Seattle, Washington; 4 Center of Excellence in Substance Addiction Treatment and Education, VA Puget Sound Health Care System, Seattle, Washington; 5 Department of Emergency Medicine, Columbia University Irving Medical Center, New York, New York; 6 Department of Epidemiology, Columbia University Mailman School of Public Health, New York, New York; 7 San Francisco VA Medical Center, San Francisco, California; 8 Department of General Internal Medicine, University of California, San Francisco; 9 Department of Population Health, Center for Opioid Epidemiology and Policy, New York University Langone Health, New York; 10 Department of Health Systems and Population Health, School of Public Health and Community Medicine, University of Washington, Seattle; 11 Department of Psychiatry & Behavioral Sciences, University of Washington School of Medicine, Seattle; 12 Department of Epidemiology, Yale University School of Public Health, New Haven, Connecticut; 13 Department of Internal Medicine, Yale University School of Medicine, New Haven, Connecticut

## Abstract

**Importance:**

Given the personal and social burdens of opioid use disorder (OUD), understanding time trends in OUD prevalence in large patient populations is key to planning prevention and treatment services.

**Objective:**

To examine trends in the prevalence of OUD from 2005 to 2022 overall and by age, sex, and race and ethnicity.

**Design, Setting, and Participants:**

This serial cross-sectional study included national Veterans Health Administration (VHA) electronic medical record data from the VHA Corporate Data Warehouse. Adult patients (age ≥18 years) with a current OUD diagnosis (using *International Classification of Diseases, Ninth Revision, Clinical Modification [ICD-9-CM]* and *International Statistical Classification of Diseases, Tenth Revision, Clinical Modification [ICD-10-CM]* codes) who received outpatient care at VHA facilities from January 1, 2005, to December 31, 2022, were eligible for inclusion in the analysis.

**Main Outcomes and Measures:**

The main outcome was OUD diagnoses. To test for changes in prevalence of OUD over time, multivariable logistic regression models were run that included categorical study year and were adjusted for sex, race and ethnicity, and categorical age.

**Results:**

The final sample size ranged from 4 332 165 to 5 962 564 per year; most were men (89.3%-95.0%). Overall, the annual percentage of VHA patients diagnosed with OUD almost doubled from 2005 to 2017 (0.60% [95% CI, 0.60%-0.61%] to 1.16% [95% CI, 1.15%-1.17%]; adjusted difference, 0.55 [95% CI, 0.54-0.57] percentage points) and declined thereafter (2022: 0.97% [95% CI, 0.97%-0.98%]; adjusted difference from 2017 to 2022, −0.18 [95% CI, −0.19 to −0.17] percentage points). This trend was similar among men (0.64% [95% CI, 0.63%-0.64%] in 2005 vs 1.22% [95% CI, 1.21%-1.23%] in 2017 vs 1.03% [95% CI, 1.02%-1.04%] in 2022), women (0.34% [95% CI, 0.32%-0.36%] in 2005 vs 0.68% [95% CI, 0.66%-0.69%] in 2017 vs 0.53% [95% CI, 0.52%-0.55%] in 2022), those younger than 35 years (0.62% [95% CI, 0.59%-0.66%] in 2005 vs 2.22% [95% CI, 2.18%-2.26%] in 2017 vs 1.00% [95% CI, 0.97%-1.03%] in 2022), those aged 35 to 64 years (1.21% [95% CI, 1.19%-1.22%] in 2005 vs 1.80% [95% CI, 1.78%-1.82%] in 2017 vs 1.41% [95% CI, 1.39%-1.42%] in 2022), and non-Hispanic White patients (0.44% [95% CI, 0.43%-0.45%] in 2005 vs 1.28% [95% CI, 1.27%-1.29%] in 2017 vs 1.13% [95% CI, 1.11%-1.14%] in 2022). Among VHA patients aged 65 years or older, OUD diagnoses increased from 2005 to 2022 (0.06% [95% CI, 0.06%-0.06%] to 0.61% [95% CI, 0.60%-0.62%]), whereas among Hispanic or Latino and non-Hispanic Black patients, OUD diagnoses decreased from 2005 (0.93% [95% CI, 0.88%-0.97%] and 1.26% [95% CI, 1.23%-1.28%], respectively) to 2022 (0.61% [95% CI, 0.59%-0.63%] and 0.82% [95% CI, 0.80%-0.83%], respectively).

**Conclusions and Relevance:**

This serial cross-sectional study of national VHA electronic health record data found that the prevalence of OUD diagnoses increased from 2005 to 2017, peaked in 2017, and declined thereafter, a trend primarily attributable to changes among non-Hispanic White patients and those younger than 65 years. Continued public health efforts aimed at recognizing, treating, and preventing OUD are warranted.

## Key Points

**Question:**

How did the prevalence of opioid use disorder (OUD) among patients in the Veterans Health Administration change from 2005 to 2022?

**Findings:**

In this cross-sectional study of patients in the Veterans Health Administration that included approximately 5 million adults per year, the prevalence of OUD increased from 2005 to 2017, peaked in 2017 at 1.16%, and declined until 2022 to 0.97%.

**Meaning:**

The findings suggest that the prevalence of OUD is declining, although continued public health efforts aimed at recognizing, treating, and preventing OUD are warranted.

## Teaser

This cross-sectional study uses Veterans Health Administration (VHA) data from 2005 to 2022 to examine trends in the prevalence of opioid use disorder overall and by age, sex, and race and ethnicity among VHA facility outpatients.

## Introduction

Opioid use disorder (OUD) is a chronic relapsing disease with wide-ranging morbidity, mortality, and psychosocial harm. Worldwide, over 26 million individuals are estimated to have OUD, the highest prevalence of which is in the US.^1^ As a result, substantial public health efforts have aimed to reduce the burden of OUD, including curbs on opioid prescribing,^2,3,4,5^ implementation of prescription drug monitoring programs,^6^ expansion of opioid agonist therapies,^7,8,9,10^ and greater access to addiction treatment,^11^ among other initiatives.^12,13^

US data show consistent increases in the prevalence of OUD up to 2018.^14,15,16,17,18^ However, little is known about more recent trends given that major surveillance surveys were interrupted by the COVID-19 pandemic, precluding the ability to validly merge data from 2020 to 2021 with prior and later years.^19,20^ To our knowledge, no published studies to date included data from 2020 onward, and only 2 published studies included data as recent as 2019. The first, an analysis of veterans by Hoggatt et al,^21^ reported increases in OUD diagnoses from 2009 to 2015 followed by stable diagnoses until 2019. The second, an analysis of US adults, found that OUD rates increased from 2010 to 2014 and then stabilized and slightly declined from 2016 to 2019.^22^ In addition to a lack of recent evidence on OUD trends in the overall population, there is a dearth of evidence on OUD trends by key demographic characteristics. This is an important gap, as OUD and overdose deaths have been shown to disproportionately affect adults who are male, younger than 64 years, and non-Hispanic White,^23^ although it is unclear how OUD in these subgroups is evolving over time. Understanding more recent trends both overall and among demographic groups may therefore aid in 3 goals: (1) assessing the evolution of the current opioid crisis through the lens of OUD, (2) ascertaining whether (and the extent to which) public health efforts have been successful at curbing OUD, and (3) aiding in more effectively targeting scarce resources and messaging to the populations at greatest OUD risk.

To our knowledge, there is no source of national, population-representative data on temporal trends in OUD spanning several years before the COVID-19 pandemic and from 2020 onward. However, Veterans Health Administration (VHA) data cover these years, offering information from several million patients annually across the US. While the VHA patient population is not representative of all US adults (including higher proportions of men, White individuals, older individuals, and those with low income^24^), VHA findings on trends and risk factors have contributed to important improvements in prevention strategies and health care practices nationally and internationally^25,26^ and have generally been consistent with nationally representative data on selected substance use issues.^27,28,29,30,31^ We therefore examined trends in the prevalence of OUD overall and by sex, age, and race and ethnicity using electronic medical record (EMR) data from the VHA between 2005 and 2022.

## Methods

### Sample

For this serial cross-sectional study, data from the VHA national EMR from January 1, 2005, to December 31, 2022, were obtained through the VHA Corporate Data Warehouse, a data repository for all documented care provided at a VHA facility or paid for by the VHA, including patient demographic data and diagnostic codes. The study was approved by institutional review boards at the Veterans Affairs (VA) Puget Sound, the VA New York Harbor Healthcare System, and New York State Psychiatric Institute. Given the nature of the EMR data analyzed, a waiver of informed consent was granted by the institutional review boards. This article was produced in accordance with the Strengthening the Reporting of Observational Studies in Epidemiology (STROBE) reporting guideline for cross-sectional studies.

All veterans who received outpatient VHA care across the US (≥1 primary care, emergency department, inpatient, or outpatient medical visit or mental health clinic encounter) from 2005 to 2022 were identified, with 18 yearly datasets created. Patients were excluded in a given year if they received hospice or palliative care or resided outside the 50 US states or Washington, DC. Missing and/or excluded data were minimal. A detailed sample breakdown of excluded patients in 2022 is given in eTable 1 in Supplement 1.

### Measures

The primary study outcome was an *International Classification of Diseases, Ninth Revision, Clinical Modification (ICD-9-CM)* or *International Statistical Classification of Diseases, Tenth Revision, Clinical Modification (ICD-10-CM)* diagnosis code for OUD. The *ICD* diagnostic system transitioned from *ICD-9-CM* to *ICD-10-CM* during the last quarter of 2015; thus, *ICD-9-CM* codes were used from January 1, 2005, through September 30, 2015, and *ICD-10-CM* codes were used from October 1, 2015, through December 31, 2022. *ICD-9-CM* codes included 305.5X for nondependent opioid abuse and 304.0X for opioid-type dependence. *ICD-10-CM* codes included F11.1X for opioid abuse and F11.2X for opioid dependence. Patients were coded as having an OUD diagnosis in a given year if they received 1 or more diagnoses from a health care practitioner during either outpatient or inpatient care at any VHA facility or community setting paid for by the VHA. Codes for OUD in remission were excluded (*ICD-9-CM* 304.03 and 305.53; *ICD-10-CM* F11.11 and F11.21).

Demographic characteristics included sex (men, women), age group (<35, 35-64, and ≥65 years), and race and ethnicity collected via self-report (Hispanic or Latino, non-Hispanic Black, non-Hispanic White, other race or multiracial, and unknown). Other race included American Indian or Alaska Native, Asian, and Native Hawaiian or Other Pacific Islander.

### Statistical Analysis

Prevalence of OUD was calculated as the number of patients diagnosed with OUD in a particular calendar year divided by the total number of eligible patients in that year. To test for changes in OUD over time, multivariable logistic regression models were run that included categorical study year and were adjusted for sex, race and ethnicity, and categorical age (<35, 35-64, or ≥65 years), consistent with prior studies using these data and examining trends in substance use.^15,18,30,32^ Due to lack of patient-identifying codes, direct statistical control for this was not possible. By ignoring within-person correlation over time, our results were conservative in that the estimates had wider 95% CIs than would occur if the repeated nature of the measures was statistically incorporated. Hence, given the large sample size, we found no limitations to using the more conservative estimates of variability.

Trends were examined separately for 2 periods: 2005 to 2017 and 2017 to 2022. First, we analyzed changes in the estimated prevalence of OUD diagnosis overall from 2005 to 2017 and from 2017 to 2022. Second, we examined whether trends differed by sex, age category, and race and ethnicity. Separate models were run for each of the demographic variables of interest (sex, race and ethnicity, and categorical age), including categorical study year, demographic variable of interest, and an interaction term for the demographic variable and study year, adjusting for the 2 remaining demographic variables. The estimated prevalence of OUD diagnosis (ie, proportions adjusted for age, sex, and race and ethnicity) in each year, changes in the prevalence, differences in trends between demographic subgroups, and associated 95% CIs were obtained from the margins command in Stata, version 17 (StataCorp LLC) based on the fitted logistic regression models.

Although we used a cut point of 2017 for statistical analyses, other studies and datasets have suggested slightly different years, including 2016 (as suggested by a National Survey on Drug Use and Health [NSDUH] study^22^) and 2018 (as suggested by raw-data tables from the Treatment Episode Data Set [TEDS]^33^). The year 2016 also marked the first full year of transition from use of *ICD-9-CM* to *ICD-10-CM* for coding OUD in the VHA. Two sensitivity analyses, therefore, used 2016 and 2018 as other potential temporal cutoffs for a statistical examination of trends in OUD diagnoses. This allowed for examination of alternative inflection points as well as separate analyses for time points before and after the VHA transitioned to using *ICD-10-CM* codes.

## Results

From 2005 to 2022, a total of 4 403 027 to 6 122 545 patients who received outpatient VHA care per year were identified. Of these patients, 7774 to 98 652 were excluded because they received hospice or palliative care and 53 270 to 66 221 because they resided outside the 50 US states or Washington, DC. Missing and/or excluded data were minimal (<3.0%). The final analytic sample included 4 332 165 to 5 962 564 VHA patients per year from 2005 to 2022. Table 1 presents the sample characteristics. The majority of included patients were men (95.0% in 2005; 89.3% in 2022); 5.0% in 2005 and 10.7% in 2022 were women. In 2005 and 2022, a total of 3.3% and 6.9%, respectively, were Hispanic or Latino; 14.0% and 18.6%, non-Hispanic Black; 78.7% and 67.1%, non-Hispanic White; 2.3% and 3.6%, other or multiracial; and 1.8% and 3.9%, unknown race. Most patients were aged 35 to 64 years (46.5% in 2005; 41.1% in 2022). Overall, the adjusted prevalence of VHA patients diagnosed with OUD (Figure, A) increased from 0.60% (95% CI, 0.60%-0.61%) to a peak of 1.16% (95% CI, 1.15%-1.17%) in 2017 (adjusted difference from 2005 to 2017, 0.55; 95% CI, 0.54-0.57 percentage points), followed by a significant decline to 0.97% (95% CI, 0.97%-0.98%) in 2022 (adjusted difference from 2017 to 2022, −0.18; 95% CI, −0.19 to −0.17 percentage points).

**Table 1.** Sample Characteristics in 2005 and 2022^a^

Characteristic	Patients, No. (%)	
	2005 (n = 4 332 160)	2022 (n = 5 688 506)
Sex		
Women	216 961 (5.0)	608 038 (10.7)
Men	4 115 199 (95.0)	5 080 468 (89.3)
Race and ethnicity		
Hispanic or Latino	140 871 (3.3)	389 457 (6.9)
Non-Hispanic Black	605 523 (14.0)	1 055 212 (18.6)
Non-Hispanic White	3 407 948 (78.7)	3 817 080 (67.1)
Other or multiracial^b^	99 714 (2.3)	206 845 (3.6)
Unknown	78 104 (1.8)	219 912 (3.9)
Age, y		
<35	187 692 (4.3)	468 729 (8.2)
35-64	2 015 248 (46.5)	2 338 600 (41.1)
≥65	2 129 220 (49.2)	2 881 177 (50.7)

^a^ Data are from the VHA Corporate Data Warehouse from January 1, 2005, to December 31, 2022.

^b^ Other included American Indian or Alaska Native, Asian, and Native Hawaiian or Other Pacific Islander.

**Figure.**
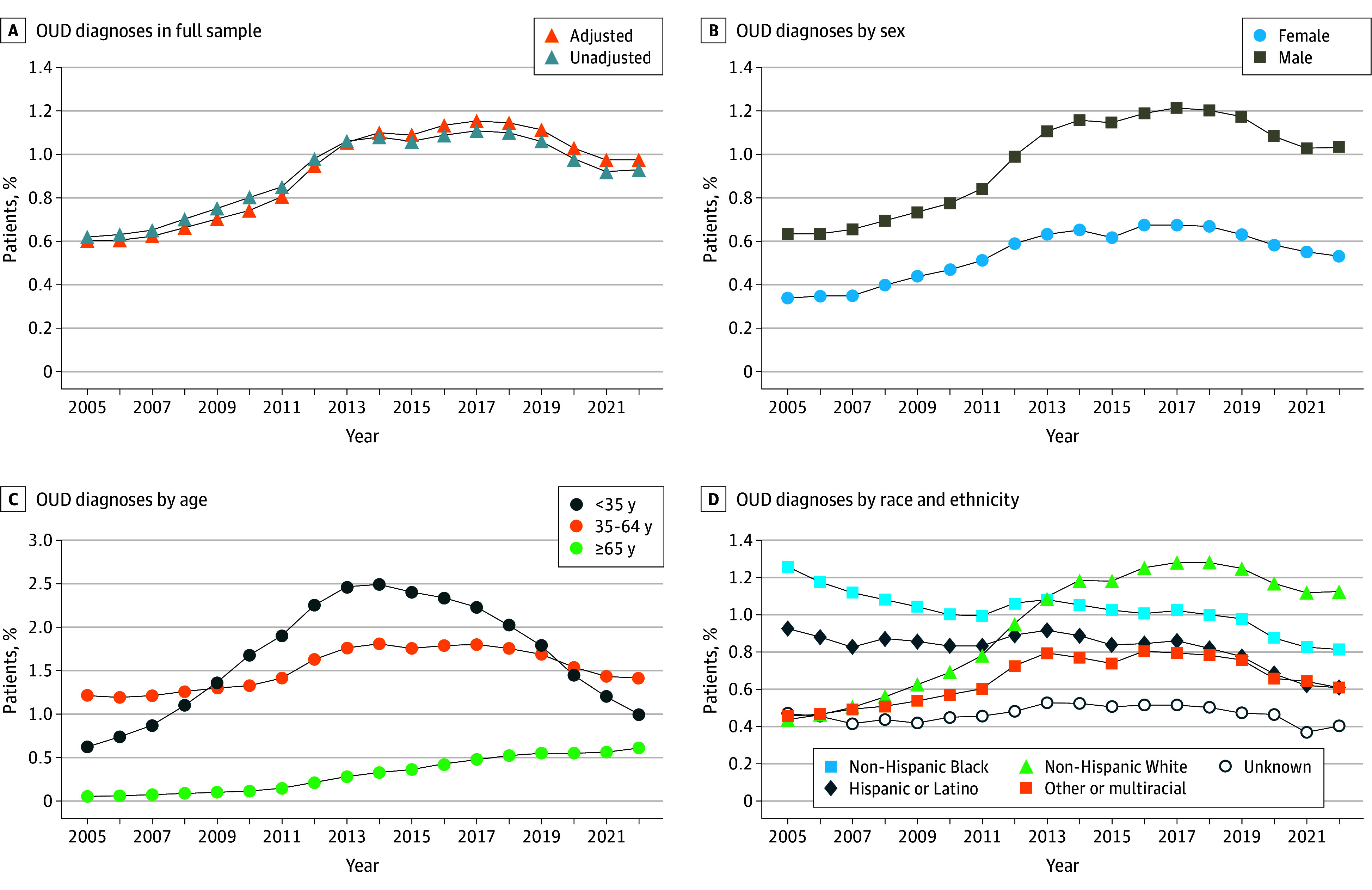
Trends in Opioid Use Disorder (OUD) Diagnoses in the Veterans Health Administration From 2005 to 2022 All models included year as the primary variable and were adjusted for sex, age, and race and ethnicity.

### Trends by Sex

Among men (Figure, B), the adjusted prevalence of OUD diagnoses increased from 0.64% (95% CI, 0.63%-0.64%) in 2005 to a peak of 1.22% (95% CI, 1.21%-1.23%) in 2017 (adjusted difference from 2005 to 2017, 0.58; 95% CI, 0.57-0.59 percentage points), followed by a significant decline to 1.03% (95% CI, 1.02%-1.04%) in 2022 (adjusted difference from 2017 to 2022, −0.18; 95% CI, −0.20 to −0.17 percentage points). Similar time trends were observed among women, for whom OUD diagnoses increased from a prevalence of 0.34% (95% CI, 0.32%-0.36%) in 2005 to a peak prevalence of 0.68% (95% CI, 0.66%-0.69%) in 2017 (adjusted difference from 2005 to 2017, 0.34; 95% CI, 0.31-0.37 percentage points) before declining to 0.53% (95% CI, 0.52%-0.55%) in 2022 (adjusted difference from 2017 to 2022, −0.14; 95% CI, −0.17 to −0.12 percentage points) (Table 2). Although rates of change were somewhat faster among men compared with women (difference in change from 2005 to 2017, 0.24 [95% CI, 0.21-0.28] percentage points; from 2017 to 2022, −0.04 [95% CI, −0.07 to −0.01] percentage points), the estimated diagnostic prevalence of OUD among men was approximately double that among women throughout the full study period.

**Table 2.** Trends in the Adjusted Prevalence of Opioid Use Disorder Diagnoses in the Veterans Health Administration From 2005 to 2022

Sample	Prevalence (95% CI), %			Change (95% CI), percentage points^a^		Difference in change (95% CI), percentage points	
	2005	2017	2022	2005-2017	2017-2022	2005-2017	2017-2022
Overall	0.60 (0.60 to 0.61)	1.16 (1.15 to 1.17)	0.97 (0.97 to 0.98)	0.55 (0.54 to 0.57)	–0.18 (−0.19 to −0.17)	NA	NA
Age category, y							
<35	0.62 (0.59 to 0.66)	2.22 (2.18 to 2.26)	1.00 (0.97 to 1.03)	1.60 (1.54 to 1.65)	–1.22 (−1.27 to −1.17)	Reference	Reference
35-64	1.21 (1.19 to 1.22)	1.80 (1.78 to 1.82)	1.41 (1.39 to 1.42)	0.59 (0.57 to 0.62)	–1.19 (−1.21 to −1.17)	−1.00 (−1.06 to −0.94)	0.83 (0.77 to 0.89)
≥65	0.06 (0.06 to 0.06)	0.48 (0.47 to 0.48)	0.61 (0.60 to 0.62)	0.42 (0.41 to 0.42)	0.14 (0.12 to 0.15)	−1.18 (−1.24 to −1.12)	1.36 (1.30 to 1.41)
By sex							
Women	0.34 (0.32 to 0.36)	0.68 (0.66 to 0.69)	0.53 (0.52 to 0.55)	0.34 (0.31 to 0.37)	–0.14 (−0.17 to −0.12)	Reference	Reference
Men	0.64 (0.63 to 0.64)	1.22 (1.21 to 1.23)	1.03 (1.02 to 1.04)	0.58 (0.57 to 0.59)	–0.18 (−0.20 to −0.17)	0.24 (0.21 to 0.28)	−0.04 (−0.07 to −0.01)
Race and ethnicity							
Hispanic or Latino	0.93 (0.88 to 0.97)	0.86 (0.83 to 0.89)	0.61 (0.59 to 0.63)	–0.07 (−0.12 to −0.01)	–0.25 (−0.29 to −0.22)	−0.91 (−0.97 to −0.86)	−0.10 (−0.14 to −0.06)
Non-Hispanic Black	1.26 (1.23 to 1.28)	1.02 (1.00 to 1.04)	0.82 (0.80 to 0.83)	–0.24 (−0.27 to −0.21)	–0.21 (−0.23 to −0.18)	−1.08 (−1.11 to −1.05)	−0.05 (−0.08 to −0.02)
Non-Hispanic White	0.44 (0.43 to 0.45)	1.28 (1.27 to 1.29)	1.13 (1.11 to 1.14)	0.84 (0.83 to 0.86)	–0.16 (−0.17 to −0.14)	Reference	Reference
Other or multiracial^b^	0.46 (0.42 to 0.49)	0.79 (0.76 to 0.83)	0.61 (0.58 to 0.64)	0.34 (0.28 to 0.39)	–0.19 (−0.24 to −0.14)	−0.50 (−0.56 to −0.45)	−0.03 (−0.08 to 0.02)
Unknown	0.47 (0.43 to 0.51)	0.52 (0.48 to 0.55)	0.40 (0.38 to 0.43)	0.05 (0.00 to 0.10)	–0.11 (−0.15 to −0.07)	−0.79 (−0.85 to −0.74)	0.04 (0.00 to 0.09)

^a^ All models included year as the primary variable and were adjusted for sex, age, and race and ethnicity. Models examining population subgroups also included an interaction term between the demographic characteristic and year (eg, sex × year).

^b^ Other included American Indian or Alaska Native, Asian, and Native Hawaiian or Other Pacific Islander.

### Trends by Age Group

Among VHA patients younger than 35 years (Figure, C), the adjusted prevalence of OUD increased from 0.62% (95% CI, 0.59%-0.66%) in 2005 to a peak of 2.22% (95% CI, 2.18%-2.26%) in 2017, followed by a large decline to 1.00% (95% CI, 0.97%-1.03%) in 2022. Adjusted models demonstrated a significant increase in OUD from 2005 to 2017 (adjusted difference, 1.60; 95% CI, 1.54-1.65 percentage points) followed by a significant decrease from 2017 to 2022 (adjusted difference, −1.22; 95% CI, −1.27 to −1.17 percentage points). Patients aged 35 to 64 years experienced less rapid change over time, with the prevalence of OUD increasing from 1.21% (95% CI, 1.19%-1.22%) in 2005 to 1.80% (95% CI, 1.78%-1.82%) in 2017, followed by stabilization until 2017 (adjusted difference from 2005 to 2017, 0.59; 95% CI, 0.57-0.62 percentage points), and declining to 1.41% (95% CI, 1.39%-1.42%) in 2022 (adjusted difference from 2017 to 2022, −1.19; 95% CI, −1.21 to −1.17 percentage points). In contrast, among adults aged 65 years or older, OUD diagnoses increased during the entire study period, from 0.06% (95% CI, 0.06%-0.06%) in 2005 to 0.61% (95% CI, 0.60%-0.62%) in 2022 (adjusted differences from 2005 to 2017, 0.42 [95% CI, 0.41-0.42] percentage points; from 2017 to 2022, 0.14 [95% CI, 0.12-0.15] percentage points) (Table 2). Overall, the rates of change in OUD among those younger than 35 years were significantly faster than among those aged 35 to 64 years and 65 years or older from both 2005 to 2017 (difference in change for 35-64 vs ≥35 years, −1.00 [95% CI, −1.06 to −0.94] percentage points; ≥65 vs <35 years, −1.18 [95% CI, −1.24 to −1.12] percentage points) and 2017 to 2022 (difference in change for 35-64 vs ≥35 years, 0.83 [95% CI, 0.77-0.89] percentage points; ≥65 vs <35 years, 1.36 [95% CI, 1.30-1.41] percentage points).

### Trends by Race and Ethnicity

Among non-Hispanic White VHA patients (Figure, D), the adjusted prevalence of OUD increased from 0.44% (95% CI, 0.43%-0.45%) in 2005 to 1.28% (95% CI, 1.27%-1.29%) in 2017 (adjusted difference, 0.84; 95% CI, 0.83-0.86 percentage points), followed by a decline to 1.13% (95% CI, 1.11%-1.14%) in 2022 (adjusted difference, −0.16; 95% CI, −0.17 to −0.14 percentage points). Both Hispanic and non-Hispanic Black patients had the highest prevalence of OUD at the start of the study period in 2005 (Hispanic patients: 0.93% [95% CI, 0.88%-0.97%]; non-Hispanic Black patients: 1.26% [95% CI, 1.23%-1.28%]), and prevalence declined thereafter in both groups (in 2022, Hispanic patients: 0.61% [95% CI, 0.59%-0.63%]; non-Hispanic Black patients: 0.82% [95% CI, 0.80%-0.83%]). Adjusted models demonstrated consistent findings for Hispanic patients (adjusted difference from 2005 to 2017, −0.07 [95% CI, −0.12 to −0.01] percentage points; from 2017 to 2022, −0.25 [95% CI, −0.29 to −0.22] percentage points) and non-Hispanic Black patients (adjusted difference from 2005 to 2017, −0.24 [95% CI, −0.27 to −0.21] percentage points; from 2017 to 2022, −0.21 [95% CI, −0.23 to −0.18] percentage points). Trends among VHA patients with unknown race and multiracial patients showed a decline after 2017, similar to trends among non-Hispanic White patients; however, the peak prevalence and rate of decline among patients reporting other race or multiracial were significantly lower. From 2014 onward, the highest estimated prevalence of OUD was among non-Hispanic White patients followed by non-Hispanic Black and Hispanic patients and patients reporting other race.

### Sensitivity Analyses

Sensitivity analyses including changing the cutoff year from 2017 to 2016 and to 2018 yielded no changes to observed results. Full sensitivity analysis findings are given in eTables 2 and 3 in Supplement 1.

## Discussion

The current study used national data from the VHA to examine trends in the diagnostic prevalence of OUD from 2005 to 2022. Overall, the percentage of patients diagnosed with OUD peaked in 2017 at 1.16% and significantly decreased thereafter to 0.97% by 2022. This trend was similar among men, women, patients younger than 35 years, those aged 35 to 64 years, and non-Hispanic White patients. Among Hispanic and non-Hispanic Black patients, OUD diagnoses decreased from 2005 to 2022, while among patients aged 65 years or older, OUD diagnoses increased across all study years from 2005 to 2022.

Our finding that OUD diagnoses declined from 2017 onward is consistent with another study of VHA patients that found that overdose deaths peaked in 2017 and declined subsequently.^32^ These and our findings are likely due to the numerous robust efforts within and outside the VHA aimed at combatting the opioid crisis. As early as 2013, the VHA established the Opioid Safety Initiative, including programs to expand nonopioid alternatives for pain and to promote reductions in opioid prescribing.^34,35^ While declines in prescribing were not associated with reductions in fatal overdoses among VHA patients with preexisting OUD, fewer prescriptions were found to be associated with reduced incident cases of OUD among younger, more opioid-naive patients.^34^ Among those younger than 35 years, the age category least likely to have long-standing opioid prescriptions, we observed declines in OUD diagnoses starting in 2014. The VHA further implemented targeted initiatives aimed at improving access to medications for OUD in primary care.^36^ Other initiatives have aimed at using automated flagging of medical records of patients at high risk of opioid overdose, prompting a mandated case review and opening opportunities for intervention and treatment.^37,38^ These and other efforts^34,39^ have led to substantial improvements in the rate of treatment among patients diagnosed with OUD in the VHA over time,^40^ potentially accounting for the declining OUD prevalence observed in the current study.

Notably, our finding that OUD diagnoses declined from 2017 onward is similar to results from 2 general population samples, including a peer-reviewed NSDUH study^22^ and the raw, unadjusted TEDS^22,33^ data tables. It is therefore possible that in recent years, OUD has also declined outside the VHA in the general population of nonveteran adults. In 2011, the White House Office of National Drug Control Policy report was released, calling for the expansion of prescription drug monitoring programs and practitioner education initiatives, among others,^41^ leading to substantial declines in opioid prescribing nationally.^42,43^ The year 2017 was also marked by the declaration of the opioid crisis as a national public health emergency, which made available new funding for treatment and prevention^44^ and garnered substantial media coverage.^45^ Together, these efforts may have led to decreased opioid use and improved access to treatment.

It is noteworthy, however, that declining cases of OUD have occurred in the context of increasing overdose deaths, possibly owing to opioid deaths increasing primarily as a result of an increasingly toxic drug supply, including fentanyl, benzodiazepines, stimulants, and xylazine. While there were continuous increases in overdose deaths involving synthetic opioids from 2016 to 2020, there were notable declines in overdose deaths from heroin and nonsynthetic prescription opioids during the same period.^44,45^ It is also possible that individuals at the highest risk of overdose death are less likely to be diagnosed with OUD or to receive care. Future research should investigate these issues further.

Given that this study used administrative health records and therefore captured only OUD that was diagnosed, observed decreases from 2017 to 2022 may be attributable to a decline in treatment seeking. This decline may have particularly occurred during the COVID-19 pandemic in 2020 to 2021, when individuals were encouraged to isolate and restrictions prevented many addiction treatment facilities from operating as usual.^46,47^ However, since the declines were seen from 2017 onward (not 2020 onward) and were consistent with other data sources, this explanation cannot adequately account for the trend. It is also possible that in recent years, VHA physicians were capturing fewer OUD diagnoses during health care visits or documenting fewer spurious diagnoses. However, the underdiagnosis of OUD has historically exceeded overdiagnosis, but increases, not decreases, in the prevalence of OUD were recently captured by administrative records.^48^

In contrast with the overall trends and trends for most groups examined, the prevalence of OUD diagnoses decreased among Black and Hispanic VHA patients throughout the study period while the prevalence increased among VHA patients aged 65 years or older throughout the study period, including from 2017 onward. It should be noted, however, that the overall prevalence of OUD among patients aged 65 years or older consistently remained lower than in other age groups. This finding is consistent with a prior study that identified older adults as a population with one of the fastest-growing rates of OUD^15,49^ and may reflect this population’s relatively high prevalence of pain.^50^ The current study further underscored the ongoing importance of providing older adults with information, resources, accessible treatment options, and harm reduction services.

### Limitations

This study has several limitations. First, this study used *ICD-9-CM* and *ICD-10-CM* diagnosis codes from administrative record data rather than structured research assessments, leading some patients with OUD to have been missed by the current analysis. Given the large established gap between OUD in the community and the proportion of people diagnosed with OUD,^51^ there were likely large numbers of OUD cases missing from our estimates. *ICD* codes can only estimate the true prevalence of OUD, and there is no clear consensus on the best operationalization of OUD in this context. While less restrictive definitions are more likely to capture false-positive cases, more restrictive definitions (such as requiring multiple *ICD* codes over multiple health care visits) are more likely to miss true-positive cases. Given the general underdiagnosis of OUD, the current study used a less restrictive definition, requiring only 1 *ICD-9-CM* or *ICD-10-CM* diagnosis code for OUD in a given year. The impact of more restrictive definitions and non-*ICD* code–based definitions on OUD trends should be the topic of future research. A similar limitation for adults aged 65 years or older is the lack of inclusion of Medicare data, which may yield further insight into OUD diagnosis trends and should be the focus of future studies. Second, the patients in the VHA are not representative of all veterans or all adults.^24^ Still, the use of VHA data provides an opportunity to examine change over time in a patient population of millions of individuals annually, and our overall results are consistent with results of large population-representative surveys.^22,33^ Third, the VHA database does not include a variable for nonprescription opioid use; thus, trends in OUD diagnoses among patients who use illicit vs prescription opioids could not be examined.

Fourth, our analysis used 2017 as a cut point for linear modeling of time trends. This cut point was based on preliminary graphical examination of OUD prevalence over time both in the overall sample and among individual subgroups. Other datasets suggest other nearby years as potential peak points or cutoffs for fitting separate linear models, including 2016 and 2018 (suggested by the NSDUH^22^ and TEDS,^33^ respectively). Sensitivity analyses altering statistical cutoffs to 2016 and 2018 yielded no change to observed results. Fifth, due to a lack of information regarding whether individuals served in combat in the VHA health records, we were unable to control for this variable. Sixth, in October 2015, the VHA transitioned from using *ICD-9-CM* to *ICD-10-CM* codes to label OUD, potentially impacting observed trends. However, a sensitivity analysis altering the statistical cutoff to 2016 yielded no change to study results. Additionally, given prior findings that the *ICD-10-CM* tended to capture a higher prevalence of OUD,^48^ our finding of an overall decline in OUD diagnoses since 2017 may be considered conservative.

## Conclusions

This cross-sectional study found that the prevalence of OUD diagnoses among adults receiving care in the VHA increased from 2005 to 2017, peaked in 2017, and declined thereafter, a trend largely attributable to changes among non-Hispanic White patients and patients younger than 65 years. Among non-Hispanic Black and Hispanic patients, OUD diagnoses declined from 2017 to 2022, while it increased among patients aged 65 years or older in the same period. Overall, decreasing cases of OUD may reflect the success of numerous VHA and public health initiatives aimed at combating the opioid crisis that emphasized the implementation of medication-assisted OUD treatments, particularly in primary care settings. Although OUD diagnoses were found to be declining, OUD prevalence in 2022 was substantially higher than in 2005, and continued public health efforts aimed at recognizing, treating, and preventing OUD are warranted.

## Notes

Supplement 1. **eTable 1.** Sample Detailed Breakdown of the Process for Achieving the Final Analytic Sample, VHA EMR Data, 2022 **eTable 2.** Sensitivity Analysis 1: Trends in Opioid Dependence Diagnoses Using 2016 as a Cut Point **eTable 3.** Sensitivity Analysis 2: Trends in Opioid Dependence Diagnoses Using 2018 as a Cut Point

Supplement 2.
Data Sharing Statement
