# Supplementary material for: Trends in Opioid Use Disorder in the Veterans Health Administration, 2005-2022
Source: JAMA Netw Open. 2024 Dec 20;7(12):e2451821. doi: 10.1001/jamanetworkopen.2024.51821 (PMC11662256; doi:10.1001/jamanetworkopen.2024.51821)
Supplement: Supplement 1. — eTable 1. Sample Detailed Breakdown of the Process for Achieving the Final Analytic Sample, VHA EMR Data, 2022 eTable 2. Sensitivity Analysis 1: Trends in Opioid Dependence Diagnoses Using 2016 as a Cut Point eTable 3. Sensitivity Analysis 2: Trends in Opioid Dependence Diagnoses Using 2018 as a Cut Point [file jamanetwopen-e2451821-s001.pdf]

Supplemental Online Content

Gorfinkel LR, Malte CA, Fink DS, et al. Trends in opioid use disorder in the Veterans Health Administration, 2005-2022. *JAMA Netw Open*. 2024;7(12):e2451821. doi:10.1001/jamanetworkopen.2024.51821

eTable 1. Sample Detailed Breakdown of the Process for Achieving the Final Analytic Sample, VHA EMR Data, 2022

eTable 2. Sensitivity Analysis 1: Trends in Opioid Dependence Diagnoses Using 2016 as a Cut Point

eTable 3. Sensitivity Analysis 2: Trends in Opioid Dependence Diagnoses Using 2018 as a Cut Point

This supplemental material has been provided by the authors to give readers additional information about their work.

© 2024 Gorfinkel LR et al. *JAMA Network Open*

eTable 1. Sample detailed breakdown of the process for achieving the final analytic sample, VHA EMR data, 2022

	Dropped	N
Original sample		5,851,927
Patients without Veteran Flag	417	5,851,510
Possible Test Patients	67	5,851,443
Patients who died before current year	6,637	5,844,806
Patients who received hospice/palliative care	96,639	5,748,167
Patients not living in 50 states or DC	56,509	5,691,658
Patients only seen in Philippines	2,912	5,688,746
Age error	240	5,688,506
Total Excluded	163,421 (2.8%)	

© 2024 Gorfinkel LR et al. *JAMA Network Open*

eTable 2. Sensitivity Analysis 1: Trends in Opioid Dependence Diagnoses using 2016 as a cut point

	2005		2016		2022		Change 2005 to 2016		Change 2016 to 2022		2005 to 2016	2016 to 2022
	Est. %	95% CI	Est. %	95% CI	Est. %	95% CI	Est. %	95% CI	Est. %	95% CI	Difference (95% CI)	Difference (95% CI)
Full Sample	0.60	(0.60 , 0.61)	1.13	(1.12 , 1.14)	0.97	(0.97 , 0.98)	0.53	(0.52 , 0.54)	-0.16	(-0.17 , -0.15)		
By Age Category												
<35	0.62	(0.59 , 0.66)	2.33	(2.29 , 2.37)	1.00	(0.97 , 1.03)	1.71	(1.65 , 1.76)	-1.33	(-1.38 , -1.28)	Referent	Referent
35-64	1.21	(1.19 , 1.22)	1.78	(1.77 , 1.80)	1.41	(1.39 , 1.42)	0.58	(0.55 , 0.60)	-0.38	(-0.40 , -0.35)	-1.13 (-1.19 , -1.07)	0.95 (0.90 , 1.01)
>=65	0.06	(0.06 , 0.06)	0.43	(0.42 , 0.44)	0.61	(0.60 , 0.62)	0.37	(0.36 , 0.38)	0.18	(0.17 , 0.19)	-1.34 (-1.40 , -1.28)	1.51 (1.46 , 1.57)
By Sex												
Female	0.34	(0.32 , 0.36)	0.68	(0.66 , 0.70)	0.53	(0.52 , 0.55)	0.34	(0.31 , 0.37)	-0.14	(-0.17 , -0.12)	Referent	Referent
Male	0.64	(0.63 , 0.64)	1.19	(1.18 , 1.20)	1.03	(1.02 , 1.04)	0.56	(0.54 , 0.57)	-0.16	(-0.17 , -0.15)	0.22 (0.19 , 0.25)	-0.01 (-0.04 , 0.01)
By Race and Ethnicity												
Non-Hispanic White	0.44	(0.43 , 0.45)	1.25	(1.24 , 1.26)	1.13	(1.11 , 1.14)	0.81	(0.80 , 0.83)	-0.13	(-0.14 , -0.11)	Referent	Referent
Non-Hispanic Black	1.26	(1.23 , 1.28)	1.01	(0.99 , 1.02)	0.82	(0.80 , 0.83)	-0.25	(-0.28 , -0.22)	-0.19	(-0.22 , -0.17)	-1.07 (-1.10 , -1.03)	-0.06 (-0.09 , -0.04)
Hispanic/Latino	0.93	(0.88 , 0.97)	0.84	(0.81 , 0.87)	0.61	(0.59 , 0.63)	-0.08	(-0.14 , -0.03)	-0.24	(-0.27 , -0.20)	-0.90 (-0.95 , -0.84)	-0.11 (-0.15 , -0.07)
Other/Multiple	0.46	(0.42 , 0.49)	0.81	(0.77 , 0.85)	0.61	(0.58 , 0.64)	0.35	(0.29 , 0.41)	-0.20	(-0.25 , -0.15)	-0.46 (-0.52 , -0.41)	-0.07 (-0.12 , -0.02)
Unknown	0.47	(0.43 , 0.51)	0.51	(0.48 , 0.55)	0.40	(0.38 , 0.43)	0.05	(-0.01 , 0.10)	-0.11	(-0.15 , -0.07)	-0.77 (-0.82 , -0.71)	0.02 (-0.03 , 0.06)

© 2024 Gorfinkel LR et al. *JAMA Network Open*

eTable 3. Sensitivity Analysis 2: Trends in Opioid Dependence Diagnoses using 2018 as a cut point

	2005		2018		2022		Change 2005 to 2018		Change 2018 to 2022		2005 to 2018		2018 to 2022	
	Est. %	95% CI	Est. %	95% CI	Est. %	95% CI	Est. %	95% CI	Est. %	95% CI	Difference (95% CI)		Difference (95% CI)	
Full Sample	0.60	(0.60 , 0.61)	1.15	(1.14 , 1.15)	0.97	(0.97 , 0.98)	0.54	(0.53 , 0.55)	-0.17	(-0.18 , -0.16)				
By Age Category														
<35	0.62	(0.59 , 0.66)	2.02	(1.98 , 2.06)	1.00	(0.97 , 1.03)	1.39	(1.34 , 1.45)	-1.02	(-1.07 , -0.97)	Referent		Referent	
35-64	1.21	(1.19 , 1.22)	1.75	(1.73 , 1.77)	1.41	(1.39 , 1.42)	0.54	(0.52 , 0.57)	-0.35	(-0.37 , -0.32)	-0.85	(-0.91 , -0.79)	0.68	(0.62 , 0.73)
>=65	0.06	(0.06 , 0.06)	0.52	(0.51 , 0.53)	0.61	(0.60 , 0.62)	0.46	(0.45 , 0.47)	0.09	(0.08 , 0.10)	-0.93	(-0.99 , -0.88)	1.11	(1.06 , 1.16)
By Sex														
Female	0.34	(0.32 , 0.36)	0.67	(0.65 , 0.69)	0.53	(0.52 , 0.55)	0.33	(0.30 , 0.36)	-0.14	(-0.16 , -0.11)	Referent		Referent	
Male	0.64	(0.63 , 0.64)	1.20	(1.19 , 1.21)	1.03	(1.02 , 1.04)	0.57	(0.56 , 0.58)	-0.17	(-0.19 , -0.16)	0.24	(0.21 , 0.27)	-0.04	(-0.06 , -0.01)
By Race and Ethnicity														
Non-Hispanic White	0.44	(0.43 , 0.45)	1.28	(1.27 , 1.29)	1.13	(1.11 , 1.14)	0.84	(0.83 , 0.85)	-0.15	(-0.17 , -0.14)	Referent		Referent	
Non-Hispanic Black	1.26	(1.23 , 1.28)	1.00	(0.98 , 1.02)	0.82	(0.80 , 0.83)	-0.26	(-0.29 , -0.23)	-0.18	(-0.21 , -0.16)	-1.10	(-1.13 , -1.07)	-0.03	(-0.06 , 0.00)
Hispanic/Latino	0.93	(0.88 , 0.97)	0.82	(0.79 , 0.85)	0.61	(0.59 , 0.63)	-0.11	(-0.16 , -0.06)	-0.21	(-0.25 , -0.17)	-0.95	(-1.00 , -0.90)	-0.06	(-0.09 , -0.02)
Other/Multiple	0.46	(0.42 , 0.49)	0.78	(0.74 , 0.82)	0.61	(0.58 , 0.64)	0.32	(0.27 , 0.38)	-0.17	(-0.22 , -0.12)	-0.52	(-0.57 , -0.46)	-0.02	(-0.07 , 0.03)
Unknown	0.47	(0.43 , 0.51)	0.50	(0.47 , 0.53)	0.40	(0.38 , 0.43)	0.03	(-0.02 , 0.08)	-0.10	(-0.14 , -0.06)	-0.81	(-0.86 , -0.75)	0.06	(0.01 , 0.10)

© 2024 Gorfinkel LR et al. *JAMA Network Open*
