# Supplementary material for: Trends in Opioid Use Disorder in the Veterans Health Administration, 2005-2022
Source: JAMA Netw Open. 2024 Dec 20;7(12):e2451821. doi: 10.1001/jamanetworkopen.2024.51821 (PMC11662256; doi:10.1001/jamanetworkopen.2024.51821)
Supplement: Supplement 2. — Data Sharing Statement [file jamanetwopen-e2451821-s002.pdf]

Data Sharing Statement

Gorfinkel. Trends in Opioid Use Disorder in the Veterans Health Administration, 2005-2022.
JAMA Netw Open. Published December 20, 2024. doi:10.1001/jamanetworkopen.2024.51821

Data
Data available: No
